# PoPoolationTE2: Comparative Population Genomics of Transposable Elements Using Pool-Seq

**DOI:** 10.1093/molbev/msw137

**Published:** 2016-08-02

**Authors:** Robert Kofler, Daniel Gómez-Sánchez, Christian Schlötterer

**Affiliations:** ^1^Institut für Populationsgenetik, Vetmeduni Vienna, Veterinärplatz 1, 1210 Wien, Austria

**Keywords:** transposable elements, comparative genomics, bioinformatics, next generation sequencing, Pool-Seq, comparative population genomics

## Abstract

The evolutionary dynamics of transposable elements (TEs) are still poorly understood. One reason is that TE abundance needs to be studied at the population level, but sequencing individuals on a population scale is still too expensive to characterize TE abundance in multiple populations. Although sequencing pools of individuals dramatically reduces sequencing costs, a comparison of TE abundance between pooled samples has been difficult, if not impossible, due to various biases. Here, we introduce a novel bioinformatic tool, PoPoolationTE2, which is specifically tailored for the comparison of TE abundance among pooled population samples or different tissues. Using computer simulations, we demonstrate that PoPoolationTE2 not only faithfully recovers TE insertion frequencies and positions but, by homogenizing the power to identify TEs across samples, it provides an unbiased comparison of TE abundance between pooled population samples. We anticipate that PoPoolationTE2 will greatly facilitate the analysis of TE insertion patterns in a broad range of applications.

## Introduction

Transposable elements (TEs) are short stretches of DNA that selfishly propagate within genomes and are thought to be involved in diverse phenomena ranging from human diseases (Kazazian, 1998) to genome evolution (Kazazian, 2004).

Many questions about the biology of TEs can be only addressed by comparing the TE abundance among different samples, such as the activity of TEs in mutation accumulation lines (e.g., base population vs. mutated lines), the dynamics of TE invasions during experimental evolution (e.g., evolved populations at different time points), the contribution of TEs to local adaptation (e.g., populations from different areas), the evolution of TE activity (e.g., populations from different species), and the extend of somatic TE activity (e.g., different tissues) (González et al. 2008; Perrat et al. 2013; Kofler et al. 2015b). Sequencing individuals (cells) separately is either too costly or, as in the case of tissues, technically too challenging (sequencing of single cells). Sequencing pools of individuals (Pool-Seq) offers a viable alternative approach (Schlötterer et al. 2014). However, a comparison of TE abundance between pooled samples is difficult as the read depth is usually not high enough to identify all TEs within a pool. This leads to an obvious bias, with more TEs being found in the sample with more mapped reads. Although it is possible to standardize the number of reads in the samples, small differences in sequencing library preparation may introduce some additional biases: 1) insert sizes may vary between samples, with longer insert sizes leading to a higher power to identify TEs, 2) coverage heterogeneity may vary among samples (e.g., due to different DNA polymerases), and 3) genome sizes may differ between samples (e.g., due to different TE contents), where larger genomes result in lower coverage and thus fewer detected TEs. We address these problems by introducing a new data format, the physical pileup track. Analogous to the pileup track, which summarizes for every genomic site the base calls, the physical pileup summarizes the structural states (e.g., TE insert presence or absence) for every genomic site. Based on the physical pileup, our new software tool PoPoolationTE2 homogenizes the physical coverage across samples and thus also the power to identify TEs.

## PoPoolationTE2

PoPoolationTE2 is a fast and user friendly tool for analyzing TE insertions in one or more samples, where samples could be tissues, pooled individuals, or separately sequenced individuals. PoPoolationTE2 does not rely on a set of annotated TE insertions in the reference genome, thus both novel (insertions not present/annotated in the reference genome) and annotated TE insertions can be identified. Nested insertions and insertions from uncharacterized TE families, however, cannot be identified. In contrast to its predecessor PoPoolationTE (Kofler et al. 2012), PoPoolationTE2 is designed to compare TE abundance among multiple samples in one joint analysis. PoPoolationTE2 is substantially faster than its predecessor, as it is implemented in Java and uses bam files as input. Although PoPoolationTE2 was primarily designed for Pool-Seq data, it can also be used for sequenced individuals where the population frequency may serve to identify heterozygous insertions or to estimate the penetrance of somatic insertions. However, for identifying TE insertions in sequenced individuals multiple dedicated tools are available (T-Lex2 [Fiston-Lavier et al. 2015], RetroSeq [Keane et al. 2013], Jitterbug [Hénaff et al. 2015], and TE-Tracker [Gilly et al. 2014]). PoPoolationTE2 requires paired end data for at least one sample, a reference genome and either a set of TE sequences or a TE annotation. Although PoPoolationTE2 accounts for heterogeneity in sequence coverage, the number of chromosomes contributing to the pools should be similar among samples or much larger than the coverage in each sample (minimizing multiple sampling from one individual at a given genomic position).

**Fig. 1.**
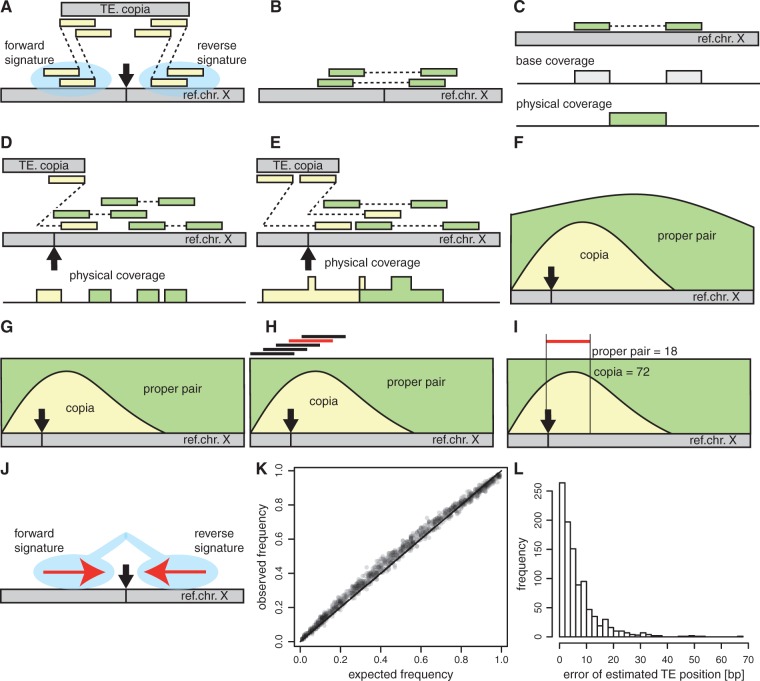
Overview of PoPoolationTE2. (*A*) TE insertions (black arrow) result in paired ends (yellow), with one read mapping to a reference chromosome (X) and the other one to a TE (copia). One group of such discordantly mapped reads is located to the left of the insertion (forward signature) and one to the right (reverse signature). (*B*) The absence of TE insertions results in proper pairs spanning a putative insertion site (green). (*C*) Mapped paired end reads may be used to generate a base coverage track (gray) and a physical coverage track (green). For the base coverage, the position of the reads is considered whereas for the physical coverage the region between the reads. (*D*) TE insertions result in paired ends that support a TE insertion (yellow). This can be translated into an additional type of physical coverage (yellow track). The median distance of proper pairs is used to estimate the distance between such discordant pairs. (*E*) Increasing the inner distance between paired ends compared with panel D results in more reads supporting a TE insertion (copia) and a higher physical coverage. If paired ends are overlapping the physical coverage of individual-paired ends is summed up, contributing to the total height of the physical coverage track. Physical coverage supporting the presence (yellow) and absence (green) of a TE may overlap (central region). (*F*) Combining the information of all paired ends for each genomic position results in a physical coverage track. (*G*) To homogenize the power to identify TEs, the physical coverage is randomly sampled to equal levels for each genomic position. (*H*) The position of signatures of TE insertions is determined using a sliding window (black lines on top) approach and the window with the maximal physical coverage supporting a TE (the red line indicates the window with the highest copia coverage) is used for further analysis. (*I*) The population frequency of TE signatures is estimated from the ratio of average physical coverage supporting a TE to the total physical coverage in a window (copia =72/(72+18)=0.8). (*J*) Matching pairs of TE signatures (forward and reverse) of the same TE family within a given distance are joined, yielding a final set of TE insertions. Final population frequency and position estimates are obtained by averaging the estimates for forward and reverse signature. (*K*) Accuracy of the population frequency estimates for 1,000 TEs in a simulated pooled population. PoPoolationTE2 has a slight upward bias for intermediate frequency TEs and a slight downward bias for high frequency TEs. (*L*) Accuracy of insertion position estimates for 1,000 TEs in a simulated pooled population.

PoPoolationTE2 requires reads to be mapped to a modified genome, consisting of a reference genome with masked TE sequences and a set of TE sequences. Masking of TEs may be done based on a TE annotation, RepeatMasker (Smit et al. 1996-2010) or, as RepatMasker sometimes misses TE insertions (Rahman et al. 2015), iterative mapping of reads derived from TE sequences (see Manual). When reads are mapped to such a modified genome, TE insertions will result in groups of discordantly mapped paired ends, where one read maps to the reference chromosome and the other to a TE sequence (signatures of TE insertions; fig. 1A), whereas properly mapped paired ends indicate the absence of a TE insertion (fig. 1B). Based on the position of mapped paired ends, a physical pileup track is generated (fig. 1C). In contrast to base coverage, which relies on the position of reads, physical coverage is based on the sequence spanned by paired ends (fig. 1C; for the difference between base and physical coverage see also Meyerson et al. 2010). Different types of physical coverage can be distinguished. Properly mapped pairs result in coverage supporting the absence of a TE while discordantly mapped reads, with one read mapping to a reference chromosome and the other to a TE, result in coverage supporting the presence of a TE (fig. 1D). Because the distance between discordantly mapped reads is not known, we use the median of the distance between proper pairs as approximation (inferred for each sample separately). The power to identify TEs scales with the number of mapped reads as well as the distance between the reads, a property that is captured by physical coverage (fig. 1E). The physical coverage of overlapping paired ends is summed up yielding a physical coverage track with the height reflecting the number of paired ends spanning a given position (fig. 1F). The power to identify TEs can be homogenized across samples by randomly sampling the physical coverage to equal levels between and within samples (fig. 1G). Signatures of TE insertions are identified with a sliding window approach scanning for peaks in physical coverage supporting a TE insertion (fig. 1H). The population frequency of TEs is estimated as the ratio of physical coverage supporting a TE insertion to the total physical coverage (fig. 1I). Finally, pairs of TE signatures (forward and reverse) of the same family within a given distance are joined (fig. 1J). PoPoolationTE2 reports the position, the family, the strand, and the population frequency for every TE in all samples. A more detailed explanation of the PoPooaltionTE2 algorithm can be found in the manual (https://sourceforge.net/p/popoolation-te2/wiki/Home/, last accessed August 8, 2016).

## Performance

First, we assessed the performance of PoPoolationTE2 under optimal conditions such that in principle all TEs could be detected. We simulated a population of size *N* = 100 with 1,000 TE insertions, used a minimum distance of 990 bp between insertions and randomly picked the family, the strand, and the population frequency (0.01≤f≤1.0) of the TEs (total size of one genome ≈3.3 Mb). For this population, we simulated paired end reads with an uniform genomic distribution and a coverage sufficiently high to detect all TE insertions (average physical coverage in the pool ≈200). The mapping algorithm may have a substantial influence on the identification of TEs. Evaluating the suitability of different alignment algorithms, we found that local alignments, with only a fraction of the read required to match, perform consistently better than semiglobal algorithm, with the entire read matching (supplementary table S1, Supplementary Material online). All local alignment algorithm tested (bwa bwasw, bwa mem, bowtie2–local [Li and Durbin, 2009, 2010; Langmead and Salzberg, 2012]) allowed for a robust identification of TEs even with sequencing error/polymorphism rates up to 10–15% (supplementary table S1, Supplementary Material online). The best results were obtained when we aligned both reads of paired ends separately using bwa bwasw and then restored the paired end information with PoPoolationTE2 (supplementary table S1, Supplementary Material online). We used this approach for the remaining analyses. We reasoned that variation of the inner distance (= fragment size–2 * read length) may cause problems with mapping strategies relying on paired ends. Consistent with this hypothesis, small variation in fragment sizes yields the most accurate estimates of the population frequency and of TE positions (supplementary table S2, Supplementary Material online). The accuracy slightly decreases with increasing variation of the inner distance (supplementary table S2, Supplementary Material online). The physical coverage derived from paired ends only depends on the mapping position of the reads. Hence, we evaluated how the sequencing strategy could be optimized to obtain the highest accuracy at the lowest sequencing costs. As long as mapping positions are not altered, the cost of sequencing may be reduced by shorter reads. Optimal results were obtained with reads of 75–100 bp length (supplementary table S3, Supplementary Material online) but decreasing the read length further than 50–75 bp misses many TEs (false negatives; supplementary table S3, Supplementary Material online). Interestingly, increasing the read length improves the accuracy of the TE position but decreases the accuracy of the population frequency estimates (supplementary table S3, Supplementary Material online). The physical coverage, and thus the power to identify TEs, scales with the number of reads and the inner distance. Thus, the cost of sequencing may be reduced by sequencing fewer reads with longer inner distances. When varying both parameters such that the physical coverage remains constant, we found the highest accuracy with inner distances between 75 bp and 200 bp (supplementary table S4, Supplementary Material online). Further increase of the inner distance may lead to inaccurate TE positions and to more false negative TEs (supplementary table S4, Supplementary Material online). For an overview of the performance of PoPoolationTE2 under optimal conditions, see table 1.

**Table 1.** Performance of PoPoolationTE2 under optimal conditions such that, in principle, all TEs could be identified. We evaluated the influence of sequencing error rate, inner distance between paired ends (ID), standard deviations of the inner distance (*σ_ID_*), read length, and the product between read numbers and inner distance (keeping the physical coverage constant). The performance was assessed by the number of identified TEs, missed TEs, false positive TEs, TEs with correct strand (strand), TEs with both signatures identified (both sign.), and TEs with a single signature identified (one sign.). Furthermore, we assessed the accuracy of the estimated insertion positions (mean: $\mu_{\Delta pos}$, standard deviation: $\sigma_{\Delta pos}$) and of the estimated population frequencies (mean: $\mu_{\Delta freq}$, standard deviation: $\sigma_{\Delta freq}$). The resulting average coverage (*μ_c_*) and average physical coverage in the pool (*μ_pc_*) were estimated from the data.

	Error Rate	Error Rate	*σ_ID_*	*σ_ID_*	Read Length	Read Length	Reads* ID	Reads* ID
Error rate	0%	10%	0%	0%	0%	0%	0%	0%
Reads [million]	6.58	6.58	6.58	6.58	6.58	6.58	13.16	3.29
ID	100	100	100	100	100	100	50	200
*σ _ID_*	20	20	0	75	20	20	20	20
Read length	100	100	100	100	50	200	100	100
*μ_c_*	394.8	317.1	395.1	395.1	198.0	780.5	790.3	197.6
*μ_pc_*	193.0	109.2	199.9	187.8	188.0	191.8	191.1	196.1
Found	999	994	1,000	998	991	1,000	1,000	996
Missed	1	6	0	2	9	0	0	4
False positive	4	10	5	8	20	2	10	6
Strand	999	994	1,000	996	988	998	995	993
Both sign.	996	982	1,000	990	986	998	996	986
Single sign.	3	12	0	8	5	2	4	10
$\mu_{\Delta pos}$	4.0	5.5	2.0	5.2	3.0	2.3	1.8	4.8
$\sigma_{\Delta pos}$	4.0	5.1	4.6	6.4	3.2	3.9	2.7	5.9
$\mu_{\Delta freq}$	0.030	0.029	0.019	0.043	0.021	0.079	0.092	0.020
$\sigma_{\Delta freq}$	0.016	0.022	0.009	0.023	0.010	0.036	0.042	0.017

Next, we evaluated the performance of PoPoolationTE2 with simulated Pool-Seq data. We again simulated a population of size *N*   =  100 having 1,000 TE insertions with random position, family, strand, and population frequency (0.01≤f≤1.0). In contrast to optimal conditions, we simulated randomly distributed paired ends (resulting in a heterogeneous coverage) and additionally, to reflect properties of Illumina paired end data (Kofler et al. 2015a), introduced 1% error rate of reads and 2% chimeric reads (reads derived from unrelated genomic positions). Allele frequencies are estimated with a precision of  ±2.5% and the insertion position differs on average by 7.2 bp (table 2). The population frequency of segregating insertions is slightly overestimated whereas the frequency of fixed insertions is slightly underestimated (fig. 1E). About 80% of the estimated TE positions are within 10 bp of the true position (fig. 1F), with low frequency insertions contributing most to the error in the position estimate (supplementary table S5, Supplementary Material online). The number of identified TEs decreases with the physical coverage (supplementary table S2, Supplementary Material online).

**Table 2.** Performance of different tools for identifying TEs with simulated Pool-Seq data. Randomly distributed paired end reads were simulated (2×100bp; inner distance was drawn from a normal distribution with mean 100 and a standard deviation of 20) with an error rate of 1% and 2% chimeric reads. We evaluated the performance of PoPoolationTE2 (Po.TE2), PoPoolationTE (Po.TE) (Kofler et al. 2012), and TEMP (Zhuang et al. 2014). For each tool, we used several minimum thresholds (either minimum count [mc] or minimum support [ms]). For an explanation of the evaluated parameters see table 1.

	Po.TE2	Po.TE2	Po.TE	Po.TE	TEMP	TEMP	TEMP
Threshold	mc2	mc3	mc3	mc4	ms4	ms7	ms10
Found	999	994	999	995	994	992	983
Missed	1	6	1	5	6	8	17
False positive	49	5	41	4	407	193	148
Strand	998	993	0	0	980	978	969
Both sign.	993	985	993	986	991	990	981
Single sign.	6	9	6	9	3	2	2
$\mu_{\Delta pos}$	7.2	7.2	17.8	17.8	4.3	4.1	4.0
$\sigma_{\Delta pos}$	7.6	7.6	13.1	13.0	14.0	13.0	13.0
$\mu_{\Delta freq}$	0.025	0.025	0.021	0.021	0.018	0.019	0.019
$\sigma_{\Delta freq}$	0.019	0.019	0.016	0.016	0.032	0.032	0.033
Time (min)	4.0	3.9	15.5	15.6	228.4	228.4	228.4

The performance of PoPoolationTE2 for Pool-Seq data from a single population is similar to other tools dedicated to TE analysis in pooled samples (TEMP [Zhuang et al. 2014], PoPoolationTE [Kofler et al. 2012], table 2). PoPoolationTE2, however, required the least computation time which facilitates the analysis of multiple samples. Due to different algorithm for identifying TEs PoPoolationTE2 and PoPoolationTE have a slightly different sensitivity with a given minimum count threshold (table 2; supplementary fig. S1, Supplementary Material online). We made the benchmarking data publicly available to facilitate testing other tools for TE identification using Pool-Seq data (https://sourceforge.net/p/popoolation-te2/wiki/TE-Benchmark/, last accessed August 8, 2016). The advantage of these simulated data is that the true insertions and population frequencies are known which permits to estimate the accuracy, sensitivity, and specificity of tools. Alternatively, it has been suggested to compare the performance of tools with real data using a standardized data set (Ewing, 2015). A weakness of this approach is that a good agreement between tools does not necessarily mean a high performance as all evaluated tools may be biased. Additionally in the case of discordant results between tools, it is not possible to assess which one actually performs best.

Finally, we compared the performance of PoPoolationTE2 to its predecessor, PoPoolationTE, using real Pool-Seq data from a natural *Drosophila melanogaster* population sampled 2008 in Northern Portugal (Kofler et al. 2012) and found that the two tools yield very similar results (supplementary fig. S2, Supplementary Material online).

Because PoPoolationTE2 was designed specifically for an unbiased comparison of TE abundance among samples, we tested its performance by simulating three populations with variable numbers of low frequency (*f*   =  0.01) insertions (*A*  =  1000, *B*  =  750, *C*  = 500). For each of these three populations we generated in silico different numbers of paired end reads which varied in insert sizes (table 3). With typical Pool-Seq studies only sampling a subset of the chromosomes in the sample (Schlötterer et al. 2014), it is not possible to identify all TE insertions. Nevertheless for an unbiased comparison of different samples, it is sufficient to determine the relative TE abundance. The example in table 3 shows how the analysis of the complete data set may lead to misleading results: in population A fewer TE insertions (minimum count 2) are detected than in population B, despite the opposite being true. Subsampling reads in all samples to equal numbers (i.e., identical base coverage) reduces the problem, but still causes misleading results, with population B having more insertions (table 3). Subsampling the physical coverage to equal levels in all populations consistently resulted in the least biased comparison of TE abundance between populations (table 3).

**Table 3.** Evaluating different strategies to compare TE abundance in Pool-Seq samples. We simulated three populations with different numbers of low-frequency insertions (*f* = 0.01) and paired ends with varying inner distances (ID). An unbiased comparison should result in a stable ratio between observed and simulated TEs in the three populations (i.e., a low $\sigma_{obs/sim}$). The best results were obtained when the physical coverage (p.c.) was sampled to equal levels in all three populations. Results are shown for two different minimum count thresholds (mc). The average coverage (*μ_c_*) and the average physical coverage in the pool (*μ_pc_*) were directly estimated from the data. ^a^ Coverage after sampling.

**Sampling strategy**	**Naive**	**Naive**	**Naive**	**Equal reads**	**Equal reads**	**Equal reads**	**Equal p.c.**	**Equal p.c.**	**Equal p.c.**
Population	A	B	C	A	B	C	A	B	C
Simulated TEs	1,000	750	500	1,000	750	500	1,000	750	500
ID	100	150	200	100	150	200	100	150	200
Reads (million)	1.045	1.379	2.045	1.045	1.045	1.045	1.045	1.379	2.045
*μ_c_*	199.91	266.66	399.97	199.91	202.19	204.34	199.91	266.66	399.97
*μ_pc_*	99.11	198.78	398.23	99.11	150.82	203.68	100.00^a^	100.00^a^	100.00^a^
Observed TEs (mc2)	396	676	495	396	580	455	147	64	19
Observed/simulated	0.396	0.901	0.990	0.396	0.773	0.910	0.147	0.085	0.038
$\sigma_{obs/sim}$	0.320			0.266			0.054		
Observed TEs (mc1)	784	745	496	784	742	499	469	375	251
Observed/simulated	0.784	0.993	0.992	0.784	0.989	0.998	0.469	0.500	0.502
$\sigma_{obs/sim}$	0.120			0.121			0.018		

When the inner distance of the paired end reads is similar between samples, subsampling reads to equal numbers has the same effect as homogenizing the physical coverage (supplementary table S7, Supplementary Material online), but the latter strategy identifies fewer TE insertions. PoPoolationTE2 supports both approaches.

Some applications, such as measuring TE activity in mutation accumulation lines, may depend on a reliable identification of sample specific insertions. This could be challenging as a putative absence of a TE insertion in one sample may in fact be an artefact of coverage heterogeneity. We show that coverage heterogeneity among samples may result in a substantial fraction of false sample specific insertions (supplementary table S8, Supplementary Material online). We recommend to analyze only regions with sufficient physical coverage in all samples since this dramatically reduces the number of false positives (supplementary table S8, Supplementary Material online).

We conclude that PoPoolationTE2 is a fast and user friendly tool for an unbiased comparison of TE abundance between samples, thus enabling to study TE dynamics in a broad range of applications.

## Availability

PoPoolationTE2 is implemented in Java and freely available at https://sourceforge.net/projects/popoolation-te2/ (last accessed August 8, 2016); For a detailed manual and a walkthrough using a small sample data set see https://sourceforge.net/p/popoolation-te2/wiki/Home/ (last accessed August 8, 2016). A data set for benchmarking tools for the identification of TE insertions with Pool-Seq data is available at https://sourceforge.net/p/popoolation-te2/wiki/TE-Benchmark/ (last accessed August 8, 2016).

## Supplementary Material

Supplementary figures S1 and S2 and tables S1–S8 are available at *Molecular Biology and Evolution* online (http://www.mbe.oxfordjournals.org/).

Supplementary Data
