# Supplementary material for: PoPoolationTE2: Comparative Population Genomics of Transposable Elements Using Pool-Seq
Source: Mol Biol Evol. 2016 Aug 2;33(10):2759–64. doi: 10.1093/molbev/msw137 (PMC5026257; doi:10.1093/molbev/msw137)
Supplement: Supplementary Data [file supp_33_10_2759__index.html]

PoPoolationTE2: Comparative Population Genomics of Transposable Elements Using Pool-Seq — PoPoolationTE2: Comparative Population Genomics of Transposable Elements Using Pool-Seq — Supplementary Data 

# PoPoolationTE2: Comparative Population Genomics of Transposable Elements Using Pool-Seq

## Supplementary Data

files

- Supplementary Data - pdf file
