# Supplementary material for: PoPoolationTE2: Comparative Population Genomics of Transposable Elements Using Pool-Seq
Source: Mol Biol Evol. 2016 Aug 2;33(10):2759–64. doi: 10.1093/molbev/msw137 (PMC5026257; doi:10.1093/molbev/msw137)
Supplement: Supplementary Data [file supp_msw137_suppopte2.pdf]

Supplement to PoPoolationTE2

Robert Kofler, Daniel Gomez-Sanchez and Christian Schlötterer

May 19, 2016

1 Supplementary figures

Figure 1: Differences in the algorithm between PoPoolationTE and PoPoolationTE2 lead to slightly different sensitivities with a given minimum count threshold. The figure shows a TE insertion (black arrow) in a reference chromosome and two paired end reads supporting the TE insertion, where one read maps to the reference chromosome (yellow) and the other to the TE sequence (not shown). PoPoolationTE counts the end positions of paired ends supporting the TE insertion within a given window (black bar) and PoPoolationTE2 estimates the average physical coverage in the window. Using a minimum count of 2 and the case where reads start a different positions (case 1), the TE insertion is identified with PoPoolationTE but not with PoPoolationTE2. The TE insertion is only identified by both tools when reads start at identical positions (case 2).

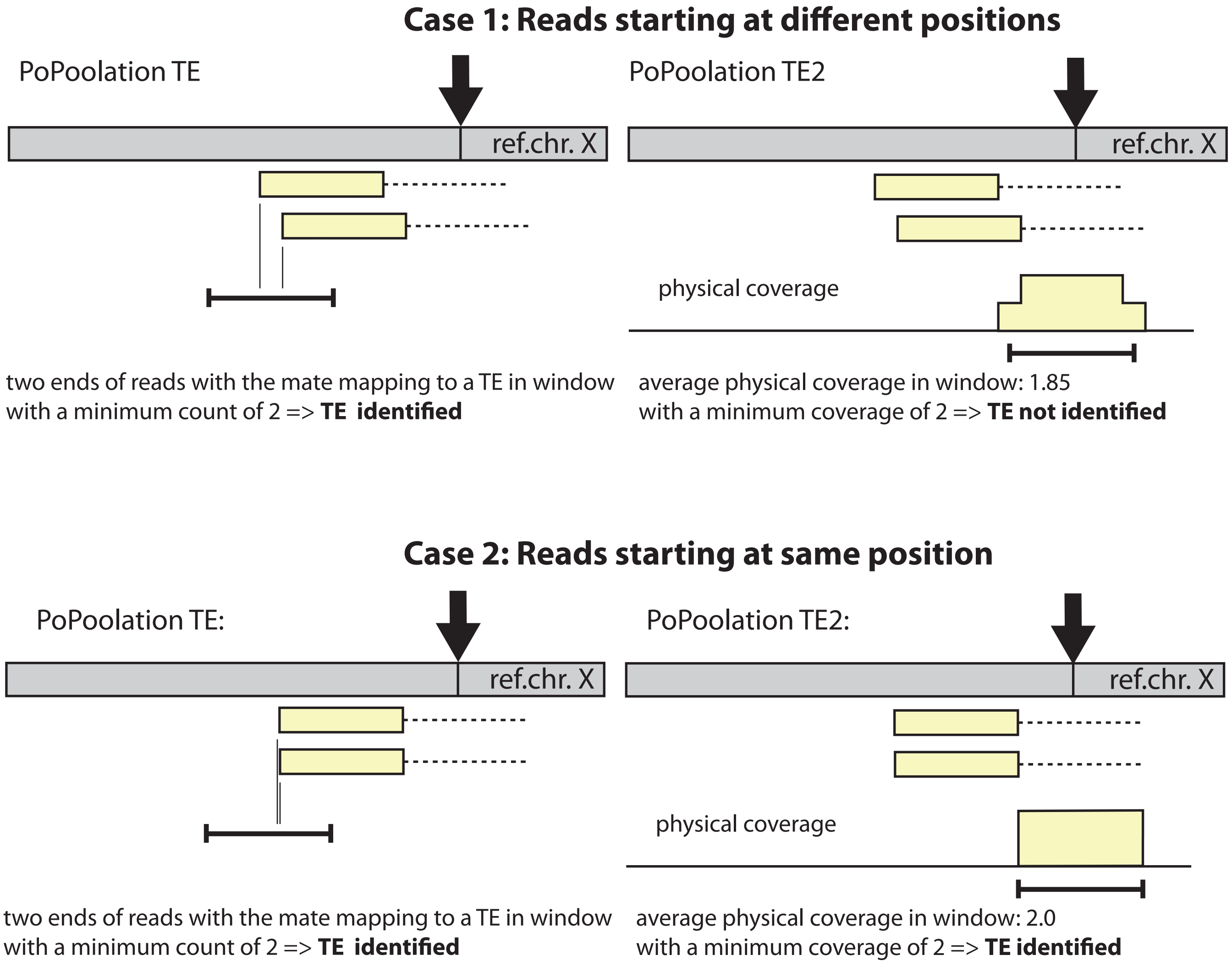

Figure 2: PoPoolationTE2 and PoPoolationTE have a similar performance using real Pool-Seq data. We obtained Pool-Seq data from a natural *D. melanogaster* population captured 2008 in Portugal (Kofler et al., 2012), mapped the reads and identified TE insertions with the two tools. We compared the abundance of insertions found for each family (A) and the overlap of the insertions with a Venn Diagram (B). Insertions were considered overlapping when both tools reported the same family and the estimated position diverged by less than 300bp. Because of slightly different sensitivities with a given minimum threshold (supplementary figure 1) we used a minimum count of 2 for PoPoolation TE2 and a minimum count of 3 for PoPoolation TE.

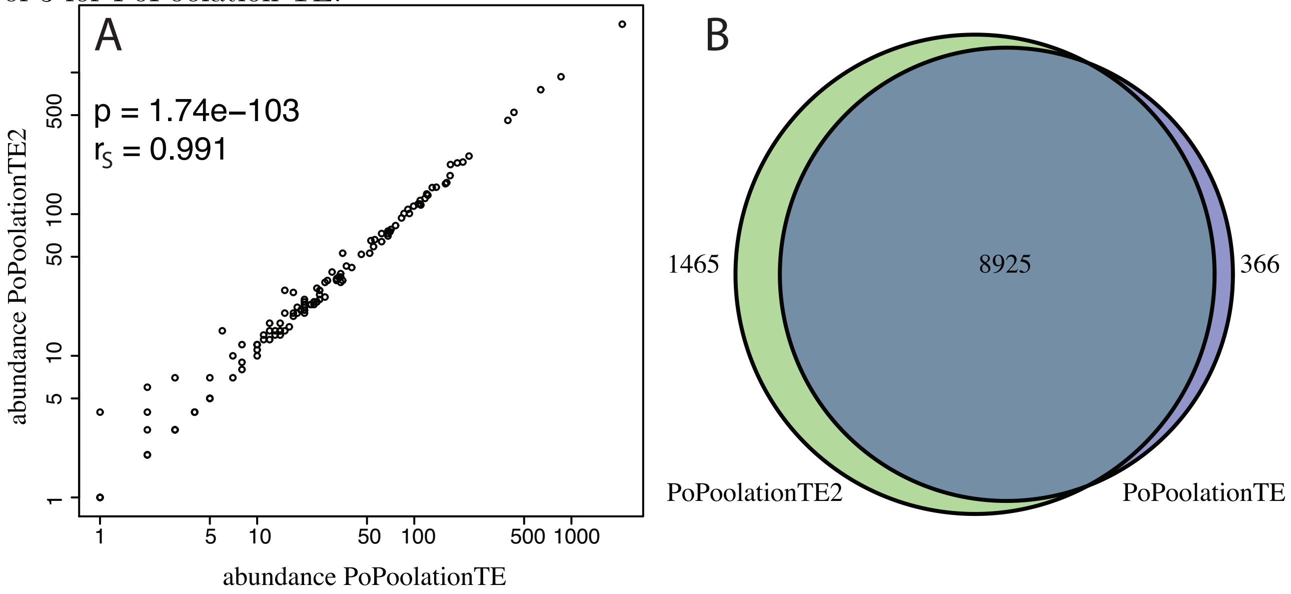

2 Supplementary tables

Table 1: caption on next page

		local alignment				semi-global alignment	
	mapper	bwa	bwa	bwa	bowtie2	bwa	bowtie2
	algorithm	se2pe	bwasw	mem	local	aln	e2e
error rate 0%	found	999	1000	1000	999	997	998
	missed	1	0	0	1	3	2
	false positive	4	8	9	5	798	10
	strand	999	996	997	998	989	992
	both sign.	996	991	987	993	986	988
	one sign.	3	9	13	6	11	10
	$\mu_{\Delta pos}$	4.0	3.8	4.7	3.0	4.1	3.0
	$\sigma_{\Delta pos}$	4.0	4.7	4.4	3.3	3.5	3.3
	$\mu_{\Delta freq}$	0.0302	0.0326	0.0370	0.0233	0.0547	0.0133
	$\sigma_{\Delta freq}$	0.0155	0.0174	0.0206	0.0200	0.0339	0.0095
error rate 5%	found	1000	1000	1000	997	987	984
	missed	0	0	0	3	13	16
	false positive	6	6	13	9	318	8
	strand	1000	1000	995	994	978	978
	both sign.	996	994	989	987	967	960
	one sign.	4	6	11	10	20	24
	$\mu_{\Delta pos}$	4.0	3.7	4.4	3.4	4.2	4.5
	$\sigma_{\Delta pos}$	4.6	4.5	4.8	4.3	4.2	4.6
	$\mu_{\Delta freq}$	0.0236	0.0146	0.0267	0.0242	0.1476	0.0500
	$\sigma_{\Delta freq}$	0.0136	0.0097	0.0156	0.0170	0.0615	0.0302
error rate 10%	found	994	994	996	990	324	253
	missed	6	6	4	10	676	747
	false positive	10	9	13	20	1	3
	strand	994	993	993	987	322	247
	both sign.	982	984	978	960	66	62
	one sign.	12	10	18	30	258	191
	$\mu_{\Delta pos}$	5.5	5.2	5.4	7.1	17.7	17.0
	$\sigma_{\Delta pos}$	5.1	5.0	5.6	7.3	12.7	11.7
	$\mu_{\Delta freq}$	0.0290	0.0568	0.0516	0.0930	0.5236	0.2051
	$\sigma_{\Delta freq}$	0.0216	0.0268	0.0221	0.0416	0.1289	0.1679
error rate 15%	found	955	955	962	902	0	0
	missed	45	45	38	98	1000	1000
	false positive	3	6	12	5	0	0
	strand	951	952	958	898	-	-
	both sign.	890	890	925	798	-	-
	one sign.	65	65	37	104	-	-
	$\mu_{\Delta pos}$	10.9	10.7	9.9	15.2	-	-
	$\sigma_{\Delta pos}$	9.8	9.8	8.6	12.8	-	-
	$\mu_{\Delta freq}$	0.0839	0.1763	0.2262	0.2334	-	-
	$\sigma_{\Delta freq}$	0.0583	0.0817	0.0938	0.1004	-	-

Table 1: Performance of PoPoolationTE2 with different alignment algorithms and sequencing error/polymorphism rates. Uniformly distributed paired end reads were simulated [2x100bp; inner distances were sampled from a normal distribution: $\mathcal{N}(\mu = 100, \sigma = 20)$]. The performance was assessed by the number of identified TEs (found), missed TEs (missed), false positive TEs (false positive), TEs with correct strand (strand), TEs with both signatures identified (both sign.) and TEs with a single signature identified (one sign.). Finally, we assessed the accuracy of the estimated insertion positions (mean: $\mu_{\Delta pos}$, standard deviation: $\sigma_{\Delta pos}$) and of the estimated population frequencies (mean: $\mu_{\Delta freq}$, standard deviation: $\sigma_{\Delta freq}$); se2pe: reads were first mapped independently with bwa bwasw and paired end information was restored using the se2pe algorithm of PoPoolationTE2

Table 2: Influence of the variation of the inner distance (σ_{ID}) on the performance of PoPoolationTE2. Uniformly distributed paired end reads were simulated [2x100bp; inner distances were sampled from a normal distribution: $\mathcal{N}(\mu = 100, \sigma = \sigma_{ID})$]. For an explanation of the benchmarks see table 1.

σ_{ID}	0	10	20	50	75	100
found	1000	1000	1000	1000	998	1000
missed	0	0	0	0	2	0
false positive	5	4	6	7	8	7
strand	1000	1000	999	998	996	998
both sign.	1000	999	997	997	990	996
one sign.	0	1	3	3	8	4
$\mu_{\Delta pos}$	2.0	2.6	3.5	4.7	5.2	5.4
$\sigma_{\Delta pos}$	4.6	3.4	4.0	4.7	6.4	7.1
$\mu_{\Delta freq}$	0.0190	0.0241	0.0302	0.0421	0.0426	0.0387
$\sigma_{\Delta freq}$	0.0087	0.0115	0.0150	0.0223	0.0234	0.0227

Table 3: Influence of the read length (RL) on the performance of PoPoolationTE2. Uniformly distributed paired end reads were simulated [2xRLbp; inner distances were sampled from a normal distribution: $\mathcal{N}(\mu = 100, \sigma = 20)$]. For an explanation of the benchmarks see table 1. Increasing the read length improves the accuracy of the TE position but decreases the accuracy of the population frequency estimates. This is likely due to more soft-clipped reads (only a part of the read aligns with the reference). With an increasing read length more reads supporting a TE insertion, will match only partly to the reference chromosome, while the other part, aligning with a TE sequence, will be clipped. With many of these reads being clipped directly at the TE insertion site, the accuracy of the estimated TE position increases. On the other hand, the physical pileup track starts directly at the clipped site, which could on the average lead, especially for long reads, to a shift in the physical pileup tracks between reads supporting a TE insertion (frequently clipped) and reads not supporting a TE insertions (unclipped). This differential phasing between pileup tracks leads to more inaccurate estimates of population frequencies with longer reads.

RL	35	50	75	100	200	500
found	0	991	1000	1000	1000	1000
missed	1000	9	0	0	0	0
false positive	0	20	7	5	2	0
strand	na	988	1000	1000	998	1000
both sign.	na	986	992	999	998	1000
single sign.	na	5	8	1	2	0
$\mu_{\Delta pos}$	na	3.0	3.1	3.5	2.3	1.2
$\sigma_{\Delta pos}$	na	3.2	3.3	4.1	3.9	3.8
$\mu_{\Delta freq}$	na	0.0209	0.0132	0.0299	0.0794	0.1854
$\sigma_{\Delta freq}$	na	0.0106	0.0084	0.0149	0.0360	0.0870

Table 4: Influence of the inner distance (ID) and the number of reads ($reads$) on the performance of PoPoolationTE2. Uniformly distributed paired end reads were simulated [2x100bp; inner distances sampled from a normal distribution: $\mathcal{N}(\mu = 100, \sigma = 20)$]. The average coverage (μ_c) and the average physical coverage (μ_{pc}) were directly estimated from the data. For an explanation of the benchmarks see table 1.

ID	25	50	75	100	150	200	400
reads [million]	26.31	13.16	8.77	6.58	4.39	3.29	1.64
μ_c	1580.67	790.32	526.87	395.14	263.42	197.57	98.77
μ_{pc}	210.85	191.06	192.10	193.60	195.22	196.10	197.30
found	1000	1000	1000	1000	1000	996	984
missed	0	0	0	0	0	4	16
false positie	6	10	5	5	6	6	5
strand	991	995	1000	1000	999	993	981
both sign.	1000	996	998	998	996	986	979
single sign.	0	4	2	2	4	10	5
$\mu_{\Delta pos}$	1.0	1.8	3.6	3.4	4.1	4.8	7.7
$\sigma_{\Delta pos}$	1.0	2.7	3.5	3.9	5.2	5.9	8.0
$\mu_{\Delta freq}$	0.1735	0.0922	0.0499	0.0301	0.0087	0.0201	0.0637
$\sigma_{\Delta freq}$	0.0792	0.0420	0.0235	0.0149	0.0059	0.0172	0.0403

Table 5: Influence of the population frequency of TE insertions on the performance of PoPoolation2 under Pool-Seq conditions [randomly distributed paired ends (2x100bp) with an error rate of 1% and 2% chimeric reads; inner distances were drawn from a normal distribution $\mathcal{N}(\mu = 100, \sigma = 20)$]. Results are plotted for different allele frequency bins (afb). For an explanation of the benchmarks see table 1.

afb	(0.0-0.1]	(0.1-0.2]	(0.2-0.3]	(0.3-0.4]	(0.4-0.5]	(0.5-0.6]	(0.6-0.7]	(0.7-0.8]	(0.8-0.9]	(0.9-1.0]
found	111	125	97	107	87	100	92	91	100	89
missed	1	0	0	0	0	0	0	0	0	0
false positive	0	0	0	0	0	0	0	0	0	0
strand	110	125	97	107	87	100	92	91	100	89
both sign.	105	125	97	107	87	100	92	91	100	89
single sign.	6	0	0	0	0	0	0	0	0	0
$\mu_{\Delta pos}$	15.6	11.7	7.5	5.8	5.1	5.8	4.8	4.8	4.0	4.1
$\sigma_{\Delta pos}$	13.1	9.2	6.2	5.1	3.5	4.0	3.8	3.7	3.0	3.0
$\mu_{\Delta freq}$	0.0117	0.0243	0.0327	0.0334	0.0345	0.0337	0.0283	0.0207	0.0127	0.0154
$\sigma_{\Delta freq}$	0.0103	0.0151	0.0181	0.0221	0.0215	0.0202	0.0183	0.0123	0.0100	0.0102

Table 6: Influence of subsampling the physical coverage (cov.) on the performance of PoPoolationTE2 under Pool-Seq conditions [randomly distributed paired ends (2x100bp) with an error rate of 1% and 2% chimeric reads; inner distances were drawn from a normal distribution $\mathcal{N}(\mu = 100, \sigma = 20)$]. Reads were mapped, the physical coverage was subsampled to the target coverage (cov.) and TEs were identified. For comparision the results without subsampling are shown (full). For an explanation of the benchmarks see table 1. As expected fewer TEs are identified with a lower coverage. However the number of identified TEs also decreases with the target coverage as the number of sites having a sufficiently coverage diminishes (only downsampling is used).

cov.	10	25	50	100	150	200	225	full
found	815	940	976	992	998	924	172	999
missed	185	60	24	8	2	76	828	1
false positive	0	1	2	5	14	17	1	49
strand	815	939	976	991	997	918	172	998
both sign.	789	922	962	984	988	859	156	993
single sign.	26	18	14	8	10	65	16	6
$\mu_{\Delta pos}$	7.2	7.3	7.2	7.2	7.1	25.2	31.4	7.2
$\sigma_{\Delta pos}$	6.3	6.8	6.9	7.5	7.8	23.5	15.9	7.6
$\mu_{\Delta freq}$	0.0313	0.0275	0.0257	0.0249	0.0245	0.0348	0.0499	0.0246
$\sigma_{\Delta freq}$	0.0224	0.0196	0.0190	0.0186	0.0185	0.0325	0.0450	0.0185

Table 7: Evaluating different strategies to compare TE abundance in Pool-Seq samples. We simulated three populations with different numbers of low frequency insertions ($f = 0.01$) and paired ends with identical inner distances (ID). An unbiased comparison should result in a stable ratio between observed and simulated TEs in the three populations (i.e. a low $\sigma_{obs/sim}$). The best results were obtained when the physical coverage (phy. cov.) was sampled to equal levels in all three populations. Results are shown for two different minimum count thresholds (mc). The average coverage (μ_c) and the average physical coverage (μ_{pc}) was directly estimated from the data. * coverage after sampling

sampling strategy	naive			equal reads			equal phy. cov.		
population	A	B	C	A	B	C	A	B	C
simulated TEs	1000	750	500	1000	750	500	1000	750	500
ID	100	100	100	100	100	100	100	100	100
reads [million]	1.045	2.067	4.090	1.045	1.045	1.045	1.045	2.067	4.090
μ_c	200.07	400.03	800.03	200.07	202.20	204.46	200.07	400.03	800.03
μ_{pc}	99.22	198.12	396.13	99.22	100.36	101.60	100.00*	100.00*	100.00*
observed TEs (mc2)	415	663	496	415	299	230	145	69	21
observed/simulated	0.415	0.884	0.992	0.415	0.399	0.406	0.145	0.092	0.042
$\sigma_{obs/sim}$		0.307			0.031			0.052	
observed TEs (mc1)	792	720	499	792	585	490	484	380	228
observed/simulated	0.792	0.960	0.998	0.792	0.780	0.98	0.484	0.501	0.456
$\sigma_{obs/sim}$		0.110			0.112			0.025	

Table 8: Evaluating strategies to identify sample specific TE insertions. We simulated two samples with different numbers of fixed ($f = 1.0$) TE insertions that where either specific to one sample (sa. sp.) or shared between samples. The fewest false positives (FP) sample specific insertions were identified when the analysis was restricted to regions having sufficient physical coverage (phy. cov.) in all samples (which was achieved by subsampling the physical coverage to 2). However, this approach also reduced the number of true positives (TP). The average coverage (μ_c) and the average physical coverage (μ_{pc}) was directly estimated from the data. * coverage after sampling; ^{a} the average coverage after subsampling is higher because only sites with sufficient coverage are retained

		naive		equal phy. cov.	
		A	B	A	B
simulated	sa.sp.	100	50	100	50
	shared	200	200	200	200
	reads	23,257	210,347	23,257	210,347
	μ_c	2.31	19.85	2.31	19.85
	μ_{pc}	1.57^a	9.89	2.00^{*a}	2.00^*
observed	TP sa. sp.	81	50	72	22
	FP sa. sp.	0	83	1	5
	shared	116	116	147	147

3 Supplementary material and methods

3.1 Performance under optimal conditions

We evaluated the performance of PoPoolationTE2 under optimal conditions such that in principle all TEs could be detected. We simulated a population of size $N = 100$ with 1000 TE insertions and generated pooled paired end sequences for this population [Pool-Seq; (Schlötterer et al., 2014)]. The simulations were performed with SimulaTE (Pandey et al; in preparation; https://sourceforge.net/projects/simulates) and involve three steps: 1) TE insertion sites within the population are defined 2) a genome is build for every individual in a population and 3) reads are directly simulated from this population genome. Finally we used PoPoolationTE2 to identify TE insertions and evaluated the performance by comparing the expected to the observed TEs.

3.1.1 Template sequence for inserting TEs

SimulaTE requires a template sequence into which TEs will be inserted. To avoid confounding effects of repetitive genomic regions in the template sequence, we evaluated the performance of PoPoolationTE2 using an artificial chromosome depleted of repetitive regions. This ensures that all reads will be unambiguously mapped to the reference genome and thus that in principle all TE insertions could be identified. We will refer to this artificial chromosome as the chassis.

To built the chassis we identified repetitive regions on chromosome 2R of *D. melanogaster* (v6.07; Flybase; (Attrill et al., 2015)) with RepeatMasker (open-4.0.3 (Smit et al., 1996-2010) using the RMBlast (v2.2.28) search engine and the settings recommended by Permal et al. (2012): -gccalc -s -cutoff 200 -no_is -nolow -norna -gff -u) and a custom repeat library consisting of the consensus sequences of Drosophila TEs [FlyBase; transposon_sequence_set.embl; v9.42; (Attrill et al., 2015; Quesneville and Anxolabéhère, 1998); we only used TE sequences found in *D. melanogaster*, *D. simulans* or *D. mauritiana*]. Masked regions were removed from the chromosome.

Finally, remaining repetitive regions were identified by creating artificial reads of size 75bp and 100bp for this chromosome, with one read starting at every base. These reads were mapped back to a modified genome consisting of the masked chromosome and the TE consensus sequences (see above) with bwa bwasw (v.7.5a) (Li and Durbin, 2010). We removed all regions from the chromosome, where reads with low mapping (mq < 50) quality aligned. We iteratively repeated this procedure (generating reads, mapping reads, removing ambiguous regions) until no further reads with low mapping quality could be found. We used the first million base pairs of this modified chromosome 2R for further analysis.

3.1.2 Artificial populations with 1000 random TE insertions

Building a population genome with SimulaTE requires a.) a template into which TE sequences will be inserted and b.) TE sequences that will be inserted. We used the chassis

(see section 3.1.1) as template and the consensus sequences of Drosophila TEs (section 3.1.1). We discarded all sequences smaller than 100bp and the Stalker4 family (due to excessive sequences similarity to Stalker3). Hence, we retained the consensus sequences of 123 TE families as final set of TE sequences (table 9). For a haploid population of size $N = 100$, we generated 1000 TE insertions with random family (table 9), strand (either sense or antisense) and population frequency, ranging from 0.01 and 1.0 (with $N = 100$ the lowest possible frequency is 1/100; SimulaTE *random-TE-insertions_freq-range.py*, *build-population-genome.py*). The minimum distance between two consecutive insertions was 990bp.

3.1.3 Simulating paired-end reads

We simulated paired end reads from a population of genomes with TE insertions (section 3.1.2) using SimulaTE. SimulaTE simulates paired ends reads having either a uniform distribution along the chromosomes (*generate-reads_paired-end-uniformdistribution.py*) or paired ends having random positions, which aims to capture the properties of Pool-Seq data (*generate-reads_paired-end.py*). To evaluate the performance of PoPoolationTE2 we simulated paired ends using a set of default parameters and varied, if not mentioned otherwise, only one parameter of interest. For uniformly distributed paired ends we used the following default parameters: read length 100, inner distance 100, standard deviation of the inner distance 20, physical coverage per haploid genome 2, error rate in the reads 0%. Randomly distributed reads were generated with default parameters: read length 100, inner distance 100, standard deviation of the inner distance, physical coverage per haploid genome 2, error rate of the reads 1% and fraction of chimeric reads 2%.

3.1.4 Identifiying TEs with PoPooaltionTE2

Unless mentioned otherwise, reads were mapped with bwa bwasw (v0.7.5) (Li and Durbin, 2010) to a modified genome consisting, of the chassis (section 3.1.1) and a set of TE consensus sequences (section 3.1.1). Paired-end information of the reads was restored using PoPoolationTE2 (*se2pe*, parameters: –sort) and a physical pileup file was created (*ppileup*) using a minimum mapping quality of 15 and a hierarchy of TE insertions extracted from the consensus sequences of TEs (see above; available from http://sourceforge.net/projects/popoolation-te2/files/publicationrelated/tehier-ml100noS4.fasta). Signatures of TE insertions were identified from the ppileup file (*identifySignatures*) with the following parameters: –mode separate, –min-count 2, the strand information of TE insertions was updated (*updateStrand*) with the parameters: –map-qual 15 –max-disagreement 0.4 and the population frequency of the signatures was estimated from the ppileup file (*frequency*). Finally, matching signatures were paired (*pairupSignatures*) using the parameters: –min-distance -200 –max-distance 300.

3.1.5 Performance with different alignment algorithms

We tested two semi-global (the the whole read is required to match) alignment algorithm, bwa aln (v0.6.2) (Li and Durbin, 2009) and Bowtie2 e2e (v2.6.2) (Langmead and Salzberg, 2012), and four local (only part of the read is required to match) algorithms, bwa bwasw (v0.7.5a) (Li and Durbin, 2010) - directly mapped as paired-end, bwa bwasw (v0.7.5a) - reads mapped as single ends and paired end information restored with PoPoolationTE2 (se2pe), bwa mem (v0.7.5a), and bowtie2 local (v2.6.2). Following previous recommendations (Kofler et al., 2011) we used for bwa aln the parameters: -o 1 -n 0.01 -l 200 -e 12. For all other tools we used default parameters.

3.1.6 Performance of different tools for identifying TEs

With PoPoolationTE2 we identified TEs as described above (section 3.1.4). To identify TEs with PoPoolationTE, both reads were mapped with bwa bwasw (v0.7.5) (Li and Durbin, 2010) to a modified reference genome consisting of the chassis (section 3.1.1) and a set of Drosophila TE sequences (section 3.1.1). Signatures of TE insertions were identified using *identify-te-insertsites.pl*, a TE hierarchy (section 3.1.4) and the parameters: –min-count 3 –narrow-range 100 –min-map-qual 15. Next, left and right TE insertion signatures were paired with *crosslink-te-sites.pl* using the parameters: –min-dist 0 –max-dist 500 –single-site-shift 100 and the population frequency was estimated with *estimate-polymorphism.pl* using the parameters: –min-map-qual 15 –te-hierarchy-level family. To identify TEs with TEMP (Zhuang et al., 2014) we mapped the reads to the chassis (section 3.1.1) with bwa aln and created a sorted bam file with samtools (Li et al., 2009). We identified TEs (TEMP v1.04; *TEMP_Insertion.sh*) using the sorted bam file and the consensus sequences of TEs (section 3.1.1). Finally we filtered for different levels of minimum support (column 6).

3.1.7 Performance of PoPoolationTE2 and PoPoolationTE using real Pool-Seq data

To validated the performance of PoPoolationTE2 with real data we obtained Pool-Seq data from a natural *D. melanogaster* population sampled 2008 in Northern Portugal [Povoa de Varzim; SRR188217; http://www.ebi.ac.uk/ena; (Kofler et al., 2012)]. Reads were mapped with bwa bwasw (v0.5.7) (Li and Durbin, 2010) to a TE-merged-reference consisting of the repeat masked reference genome of *D. melanogaster* (v5.31) and TE sequences as described in Kofler et al. (2012). The TE hierarchy was taken from (Kofler et al., 2012) (Dataset S1). TEs were identified as described above (section 3.1.4, 3.1.6). A minimum count of 3 was used for PoPoolationTE and 2 for PoPoolationTE2 (see also supplementary fig. 1)

3.2 TE abundance between samples

To evaluate the suitability of PoPoolationTE2 for comparing TE abundance between Pool-Seq samples we simulated three populations having different numbers of low frequency

($f = 0.01$) TE insertions: population $A = 1000$, population $B = 750$ and population $C = 500$ with SimulaTE. Next, we build the genomes of three populations with TE insertions (SimulaTE *build-population-genome.py*) and generated randomly distributed paired end reads as described above (section 3.1.3). Reads were mapped to the modified genome as described above (section 3.1.4) and a joint ppileup file was generated using all three populations as input (PoPoolationTE2 *ppileup*). We performed three analyses: a) we used the full set of paired ends to identify TEs b) we aimed to homogenize the power to identify TEs between the samples by subsampling all reads to equal numbers (using seqtk https://github.com/lh3/seqtk) and c) we aimed to homogenize the power to identify TEs between the samples by subsampling the physical coverage to 100 in all populations (PoPooolationTE2 *subsamplePpileup* –target-coverage 100). Finally, signatures of TE insertions were identified (PoPoolationTE2 *identifySignatures* –mode separate –min-count 2 –signature-window fix100) and suitable TE signatures were paired (PoPoolationTE2 *pairupSignatures* –min-distance -200 –max-distance 300) yielding a set of TE insertions.

To evaluate whether PoPoolationTE2 allows to identify sample-specific TE insertions we used SimulaTE to generate fixed TE ($f = 1.0$) insertions for two haploid individuals. We simulated 200 insertions shared between the two samples, 100 insertions specific to the first sample and 50 insertions specific to the second sample (supplementary table 8). We build a population genome for each sample (SimulaTE *build-population-genome.py*). Paired end reads were simulated and reads were mapped as described above (section 3.1.3). We created two ppileup files, one using the full data set (PoPoolationTE2 *ppileup*) and one by subsampling the physical coverage to 2 (PoPooolationTE2 *subsamplePpileup* –target-coverage 2). Signatures of TEs were identified (PoPoolationTE2 *identifySignatures* –mode joint –min-count 1 –signature-window fix100) and suitable signatures were paired (PoPoolationTE2 *pairupSignatures* –min-distance -200 –max-distance 300 –output-detail medium). Sample specific insertions using identified with a minimum count of 1. To ensure an unbiased comparision of the speed of the different tools we started the analysis with mapped reads for each tool (a sorted bam file). For PoPoolationTE and PoPoolationTE2 reads were mapped to the TE-merged-reference and for TEMP reads were mapped to the chassis. The time was measured by calling the date command at the beginning and the end of the analysis (using shell scripts).

3.3 Statistical analysis

The performance was assessed by comparing expected and observed TE insertions using custom Python scripts (http://sourceforge.net/projects/popoolation-te2/files/publicationrelated/scripts-popte2.zip). A TE was considered a true positive if the identified family agreed with the simulated one and the estimated position was within 200bp of the simulated position. The average base coverage was directly estimated from pileup files (samtools mpileup) and the average physical coverage from the ppileup files (PoPoolationTE2 *stat-coverage*). Statistical analyses were performed using the R programming language (v.3.1.1) (R Core Team, 2012).

Table 9: TE families used for the simulations

M14653, DME9736, DMIS176, DMTN1731, DMIS297, DM23420, 412, DMAURA, DMBARI1, BS, DMU89994, DMCOPIA, DMW1DOC, F, FB, DMTNFB, DMREPG, DMGYPF1A, DMHFL1, DMTHB1 DM06920, DMIFACA, DMLINEJA, DMTRDNA, DMRTMGD1, DMMDG3, DMDM11, PPI251, DMPOGOR11, DMRER1DM, DMRER2DM, DM33463, SPRINGER, TIRANT, DMBLPP, OPUS, DM_ROO, BLOOD, DMZAM, DME010298, ROXELEMENT, AF222049, CIRC, DME278684, RT1B, QUASIMODO, Beagle, Tinker, TABOR, STALKER, INE1, GTWIN, GYPSY2, ACCORD, 1360, GYPSY3, INVADER, INVADER2, INVADER3, G2, DMCR1A, TC1, DOC2, DOC3, IVK, RT1C, GYPSY4, INVADER4, BAGGINS, G3, MARINER2, TRANSIB1, TRANSIB3, TRANSIB2, GYPSY5, GYPSY6, INVADER5, DIVER2, TRANSIB4, S2, DM88, JUAN, FROGGER, ROVER, DMTOM1_LTR, G5_DM, G4_DM, ROOA_LTR, JOCKEY2, G6_DM, LOOPER1_DM, AF418572, QBERT, McCLINTOCK, HOPPER2, STALKER2, STALKER3, AF541951, DME487856, BS3, BS4, DOC4, DOC5, FW2, FW3, HELITRON1_DM, R1-2, TC1-2, G5A, G7, GYPSY7, GYPSY8, GYPSY9, GYPSY10, GYPSY11, GYPSY12, INVADER6, HEL, TC3, Beagle2, Q, OSV, DME542581

References

Attrill H, Falls K, Goodman JL, Millburn GH, Antonazzo G, Rey AJ, Marygold SJ, consortium F, et al. (9 co-authors). 2015. Flybase: establishing a gene group resource for drosophila melanogaster. *Nucleic acids research*. p. gkv1046.

Kofler R, Betancourt AJ, Schlötterer C. 2012. Sequencing of Pooled DNA Samples (Pool-Seq) Uncovers Complex Dynamics of Transposable Element Insertions in *Drosophila melanogaster*. *PLoS genetics*. 8:e1002487.

Kofler R, Orozco-terWengel P, De Maio N, Pandey RV, Nolte V, Futschik A, Kosiol C, Schlötterer C. 2011. Popoolation: a toolbox for population genetic analysis of next generation sequencing data from pooled individuals. *PloS one*. 6:e15925.

Langmead B, Salzberg SL. 2012. Fast gapped-read alignment with Bowtie 2. *Nature methods*. 9:357–9.

Li H, Durbin R. 2009. Fast and accurate short read alignment with Burrows–Wheeler transform. *Bioinformatics*. 25:1754–1760.

Li H, Durbin R. 2010. Fast and accurate long-read alignment with Burrows-Wheeler transform. *Bioinformatics (Oxford, England)*. 26:589–595.

Li H, Handsaker B, Wysoker A, Fennell T, Ruan J, Homer N, Marth G, Abecasis G, Durbin R. 2009. The Sequence Alignment/Map format and SAMtools. *Bioinformatics (Oxford, England)*. 25:2078–2079.

Permal E, Flutre T, Quesneville H. 2012. Roadmap for annotating transposable elements in eukaryote genomes. *Methods in molecular biology (Clifton, N.J.)*. 859:53–68.

Quesneville H, Anxolabéhère D. 1998. Dynamics of transposable elements in metapopulations: a model of P element invasion in Drosophila. *Theoretical population biology*. 54:175–193.

R Core Team. 2012. R: A Language and Environment for Statistical Computing. R Foundation for Statistical Computing, Vienna, Austria. ISBN 3-900051-07-0.

Schlötterer C, Tobler R, Kofler R, Nolte V. 2014. Sequencing pools of individuals — mining genome-wide polymorphism data without big funding. *Nature Reviews Genetics*. 15:749–763.

Smit AFA, Hubley R, Green P. 1996-2010. RepeatMasker Open-3.0.

Zhuang J, Wang J, Theurkauf W, Weng Z. 2014. TEMP: a computational method for analyzing transposable element polymorphism in populations. *Nucleic acids research*. .
